# Optimising the process of knowledge mobilisation in Communities of Practice: recommendations from a (multi-method) qualitative study

**DOI:** 10.1186/s43058-022-00384-1

**Published:** 2023-01-26

**Authors:** Laura Swaithes, Zoe Paskins, Jonathan G. Quicke, Kay Stevenson, Kathy Fell, Krysia Dziedzic

**Affiliations:** 1 Impact Accelerator Unit, Versus Arthritis Primary Care Centre, School of Medicine, Keele University, Staffordshire, ST5 5BG UK; 2 Haywood Academic Rheumatology Centre, Haywood Hospital, Midlands Partnership NHS Foundation Trust, Staffordshire, UK; 3 Haywood Hospital, Midlands Partnership NHS Foundation Trust, Staffordshire, UK; 4 Research User Group, Versus Arthritis Primary Care Centre, School of Medicine, Keele University, Staffordshire, ST5 5BG UK

**Keywords:** Community of Practice, Knowledge Mobilisation, Implementation, Musculoskeletal, Qualitative, NIHR Themed Reviews, Public contributor involvement

## Abstract

**Background:**

Communities of Practice (CoPs) offer a strategy for mobilising knowledge and integrating evidence-based interventions into musculoskeletal practice, yet little is known about their practical application in this context. This study aimed to (i) explore the process of knowledge mobilisation in the context of a CoP to implement evidence-based interventions in musculoskeletal care and (ii) co-develop recommendations to optimise the process of knowledge mobilisation in CoPs.

**Methods:**

A qualitative study comprising observation of a CoP and related planning meetings (*n* = 5), and interviews with CoP stakeholders (including clinicians, lay members, managers, commissioners, academics) (*n* = 15) was undertaken. Data were analysed using thematic analysis and interpreted considering the Integrated Promoting Action on Research Implementation in Health Services theory. Public contributors were collaboratively involved at key stages of the study.

**Results:**

Four themes were identified: identifying and interpreting knowledge, practical implementation of a CoP, culture and relationship building, and responding to the external context. Resource and infrastructure enabled the set-up, delivery and running of the CoP. Support for lay members is recommended to ensure effective participation and equity of power. CoP aims and purpose can develop iteratively, and this may enhance the ability to respond to contextual changes. Several recommendations for the practical application of CoPs are suggested to create the best environment for knowledge exchange and creation, support an equitable platform for participation, and help members to navigate and make sense of the CoP in a flexible way.

**Conclusion:**

This study identified how a CoP with diverse membership can promote partnership working at the intersection between knowledge and practice. Several important considerations for preparing for and operationalising the approach in implementation have been identified. Evaluation of the costs, effectiveness and impact of CoPs is needed to better understand the value added by the approach. More broadly, research is needed to explore the practical application of online CoPs and the role of international CoPs in optimising the uptake of innovations and best practice.

**Supplementary Information:**

The online version contains supplementary material available at 10.1186/s43058-022-00384-1.

Contributions to the literature

- Research has shown the variability in structure and function of CoPs, making it difficult to operationalise the approach. This study identifies recommendations for optimising the process of knowledge mobilisation in CoPs
- This study contributes to recognised gaps in the literature regarding public involvement in implementation e.g. ensuring that all stakeholders feel on an ‘equal footing’ at the beginning of a CoP (which may require preparation) and that lay members are clear about their role
- CoPs are a strategy for reducing healthcare boundaries and silo working and promoting partnership working and integrated services

## Background

Musculoskeletal disorders (MSKDs) are the leading cause of disability in the UK [1, 2]; costing NHS England almost £5 billion per annum [3]. The overall quality of care for MSKDs is suboptimal, partly due to the inconsistent clinical uptake of guidelines and evidence-based recommendations [4–7]. Bridging the evidence-to-practice gap to reduce clinical variation and improve the management of MSKDs is a major challenge[8]. There is a need to explore knowledge mobilisation (KM) strategies to help clinicians and other musculoskeletal stakeholders address the complexity of implementation.

Communities of Practice (CoPs) offer a strategy for mobilising knowledge into practice by working within, and across, professional, public, organisational, and policy boundaries [9–12]. By definition, a CoP is ‘a group of people, who share a concern, a set of problems, or a passion about a topic, and who deepen their knowledge and expertise in this area by interacting on an ongoing basis’ [13]. CoPs enable members to address the complexity and scale of knowledge matters within their practice by (i) sharing an understanding of a joint domain; (ii) interacting, building relationships, and negotiating meaning within the community; and (iii) over time, producing a shared repertoire of resources as they become part of the collective practice [13–17].

The varied benefits and uses of CoPs include learning from shared experiences and problem-solving [13, 14, 18]. Furthermore, the ability to create and share organisational knowledge and learn from stakeholders whose engagement is critical to success, is of central importance for implementation [19–21]. In healthcare CoPs, a shift in focus has been seen, from learning and exchanging knowledge to improving clinical practice [22]. The approach has been used for service improvement projects [23, 24], enhancing professional networking [25], improving quality standards [26], and, evaluating inter-group knowledge sharing [27]. CoPs are increasingly being used in healthcare, for example by the Health Innovation Network [28] and the NIHR East of England Applied Research Collaboration Implementation Programme [10] to enhance KM and to drive the spread of evidence-adoption and innovation.

CoPs have the potential to provide a solution to the implementation challenges facing the musculoskeletal community by bringing key stakeholders together to collaboratively identify how to take knowledge and best evidence and accelerate its uptake within local clinical services. However, uncertainty regarding potential mechanisms and processes that optimise CoPs and a lack of uniform operating definitions exist. Variation in the structure and function of CoPs can make it challenging for practitioners to utilise the approach [29, 30]. A paucity of knowledge exists regarding if, how, and why CoPs work [18, 22, 28]; and how to operationalise the approach within the field of musculoskeletal implementation [28].

The aim of this study was to explore the process of KM in the context of a CoP to implement evidence-based interventions in musculoskeletal care and to co-develop recommendations to optimise the process of KM in CoPs.

### Background overview of the moving forward implementation project

The National Institute of Health Research (NIHR) Themed Review entitled ‘Moving Forward’ [31] interprets research evidence of effective interventions for managing MSKDs, which have the potential to improve patient outcomes and reduce healthcare costs. Funding was secured from the Chartered Society of Physiotherapy (CSP) (2018) by Keele University Impact Accelerator Unit (IAU), to implement evidence from the NIHR Themed Review ‘Moving Forward’ across one county in England.

The Moving Forward implementation project was led by the IAU, an interdisciplinary knowledge brokering service, nested within a clinical academic unit of expertise [32]. The IAU comprises individuals with boundary spanning roles, offering academic and clinical leadership, project management, patient and public involvement and engagement (PPIE) and KM expertise. In addition, the team has a strong track record in researching and delivering implementation in musculoskeletal practice, change in workforce pathways and prior experience of the CoP methodology [33].

The underpinning principles of the project were informed by the KM Toolkit for Primary Care [34] (an evidence-based toolkit designed to enhance KM and implementation of clinical guidelines), with the overarching aim of supporting local musculoskeletal services to increase the use of evidence from the NIHR Themed Review ‘Moving Forward’ to optimise clinical pathways. To achieve this, a CoP was established (in 2018) with a range of clinical, commissioning, lay and academic stakeholders (including musculoskeletal clinicians, operational managers, PPIE, AHP Professional Leads, Commissioners of Services, IAU Representatives, University Partnerships Manager. Patients with lived experience of physical and mental health). All stakeholders had varied experience, expertise, perspectives, and insight relating to MSKDs and/or implementation of musculoskeletal research into practice. Membership of the CoP was via invitation from the CoP project lead (KS, Consultant Physiotherapist). Project meetings were held quarterly from Feb 2018 to Nov 2020. A dedicated website was established (https://doi.org/10.21252/dcaf-y913) by the IAU to share learnings, Vlogs, project reports, and examples of activities undertaken within the meetings. A detailed description of the CoP (including how it was established, clinical setting and some of the activities undertaken) is presented in Supplementary material Box 1.

## Methods

### Data collection and analysis

This study used two data collection methods: first, observation of CoP meetings and related planning meetings, and second, interviews with stakeholders from the CoP. Findings were used to co-develop recommendations to optimise the process of KM in CoPs. This study was reported using the Consolidated Criteria for Reporting Research checklist [35].

Observation of CoP meetings and related planning meetings were undertaken by LS (PhD, Physiotherapist and qualitative researcher) between December 2019 and January 2021. During covid-19 CoP meetings were conducted online. Observations supplemented the interview data and yielded additional insight into contextual factors affecting the CoP by providing access to rich data collected from the ‘natural’ setting of the CoP. With written consent, data were recorded as unstructured field notes which included descriptive notes and reflective memos. Written field notes included information regarding dialogue and how participants engaged and interacted with each other and CoP activities[36, 37]. These were used to identify salient features of the situation (context and process of CoP) to supplement interview data (either complementary or contradictory). Furthermore, understanding of additional concepts or new relationships was explored by iteratively adding to the topic guide[36].

Semi-structured interviews were conducted (LS) from August-December 2020, to explore the experiences and perceptions of stakeholders involved in the CoP. A purposive sampling strategy was adopted to ensure participants from a range of professional backgrounds (clinician, manager, commissioner, lay contributor) were captured [38]. Topic guides were tailored specifically to certain participant groups (e.g. lay member, clinician, non-clinical such as manager, commissioner – supplementary material 2) to facilitate the collection of a breadth and depth of relevant data. With written consent, interviews were conducted over the telephone or via MS Teams, digitally recorded and transcribed verbatim.

The anonymised interview transcripts and observation field notes were imported into NVivo 11 for analysis [39]. After a period of familiarisation, inductive coding of transcripts (LS) took place to generate initial codes and ensure that important aspects of the data were not missed [40]. The roles of the study team within the CoP were considered reflexively during data collection and analysis. LS and ZP (IAU staff members) were not involved with the CoP organisation and held several analysis meetings to discuss data before meeting with the broader study team, including KS who facilitated the CoP meetings.

Independent double coding of a sample of transcripts was then completed with co-authors (LS, KS, ZP (Consultant Rheumatologist and qualitative researcher), KD (Professor of Musculoskeletal Therapies)), and discussed in a dedicated analysis meeting. The coding was revised, and codes grouped into descriptive categories, or sub-themes, and a coding framework developed. Four further iterative cycles of constant comparison (between the coding framework, interview transcripts and observation field notes) were undertaken to refine overarching themes and subthemes. This drew on recognised techniques including the scrutiny of deviant cases, checking for confirmatory or challenging evidence within the dataset, and interpreting patterns [41]. Further analysis meetings took place after each cycle of revisions to reflect upon and discuss the themes, coding framework and illustrative quotes, and to carefully consider any connections between the empirical data, the literature and KM theory. A final coding framework was agreed and applied to the dataset.

### Co-development of recommendations

Draft recommendations for optimising the process of KM in CoPs were derived from the qualitative findings and presented at a dedicated meeting with public contributors and the study team. These were discussed, interpreted, and finalised in view of the study findings and any potential gaps. Several meetings took place between LS, public contributors, and an illustrator to co-develop a visual output of the study findings.

### Underpinning theory

The Integrated Promoting Action on Research Implementation in Health Services (i-PARIHS) theory [42] was used as underpinning theory. i-PARIHS is a useful framework for understanding the process of KM, with a prominent focus on context which we hypothesized would be important in this study exploring the context of a CoP. i-PARIHS integrates four constructs (context, innovation, facilitation, and recipients*)* commonly identified in KM literature, to facilitate evidence use within practice. I-PARIHS was used to interpret the data through a KM lens to make sense of our analysis in relation to the four constructs (Table 1). For example, by asking questions such as who are the recipients of the KM that takes place in the CoP, what are the key features of facilitation within the CoP, how does the context of the CoP influence KM? In analysis meetings, discussion included critical reflection on the insights gleaned regarding the actors and actions of KM and how the four constructs were manifest within the emerging themes.

**Table 1:** Theme descriptions and illustrative key findings

Main theme	Subtheme	Relevant i-PARIHS domains	Illustrative quote(s)
Identifying and interpreting knowledge	Types of knowledge	Innovation Context	Q1. This is an opportunity to really fully embrace the evidence (P07-PC) Obs1 (meeting 4 online). Telemedicine now important (covid pandemic). Group members presented existing research about remote consultations in msk, including a ‘virtual’ hip and knee class. Clinicians shared current clinical challenges and all seemed to find the research presented beneficial. Long group discussion about local applicability and what could be delivered in current context. Research team become aware of priorities from the ‘clinical coalface’
	Knowledge creation and use	Innovation Context Recipients	Q2. It wasn’t just patients and the public that said, ‘This document’s too complex’ it was any type of clinicians, I mean it’s quite a thick review and no one’s got time really to read through it all (P06-PNC) Q3. Being able to work alongside of people who you probably originally thought would have different thoughts to you, when it actually came together, you may have had similar ideas, but they were just put together differently but when you got a result, it was something that was mutual (P08-L)
	Negotiating knowledge	Innovation Context Recipients Facilitation	Q4. We really are just a service that gets on and does it. So we will look, ‘Okay, what’s the best evidence and who’s the best person that we’ve got?’….‘Okay, let’s get on and do it’. We can’t wait and see what’s the evidence, because we could be waiting five years and we’ve lost out (P14-PC) Q5. As much as we would love to be like, ‘Right well evidence says that you need to provide 12 sessions of treatment for this particular condition’, that’s not within our gift, because of our service specification we can’t do that. But it’s then taking the bits that we can do and then ensuring that everyone’s aware of the evidence and I think that’s where our challenge is (P11-PC) Q6. All the research that was in the Moving Forwards document they [HCPs] were all aware of anyway obviously. So, they sort of came to the community of practice with that knowledge of what they were looking at to implement in the future (P01-L) Q7. They know I’m a patient… I hadn’t got to go in with all this knowledge (P01-L) Obs2 (meeting 2, face to face). Lengthy discussion regarding current care for people with osteoarthritis. Local clinicians know that the service provided does not align with the number of sessions stated in Moving Forward research. Reluctance to alter this due to strong positive audit data. Other clinicians spoke of how the financial envelope would prevent the service delivering 12 sessions
Practical implementation of a CoP	Infrastructure and support	Facilitation Context	Q8. It’s [the CoP] one of those Swiss watch, you know it just ticks away with no whistles and bells, it just happens (P02-L) Q9. You need a strong support team underneath that lead because it’s important you don’t just go from meeting to meeting with no communication in between so, we’ve been well aware that we need to continually communicate whether that’s through newsletters, the website, bringing those members into other elements of our work (P15-PNC) Q10. The way it was organised was fantastic you know, in the diversity of the people that were there and also how they went through things. And then having clear outcomes and things to work on (P05-PC) Q11. It’s not just the project leads that have speared it, it’s everybody together and everybody has had the chance to take the floor and talk about their own context and how they can work together to drive the project forward (P06- PNC) Obs3. (meeting 3 face to face also noted meeting 4 online) The group appear to ‘turn to’ or ‘lean on’ staff from the IAU for direction, set up, support. Would the CoP function without this?
	Clarity of aims and purpose	Recipients Facilitation	Q12. Keele had already started some work, they are your best bet because they are doing it as evidence-based practice specifications and trying to make sure they are put into practice. So rather than you duplicate, you are actually working with academics who have done this and who are doing this and who have a record of actually delivering that (P04-PNC) Q13. So that’s basically how I then came onto the platform because I realised what I wanted to do was already happening elsewhere with another partner. Which is the university that they have the expertise and the skill and in a way the capacity and the manpower to be able to help that (P04-PNC) Q14. I love research, I love to try and make a difference for my patients and I believe my contribution will somehow…I can facilitate the implementation of the evidence into clinical practice…and I saw the Moving Forward idea and the work done [in the CoP] as the best vehicle to get there (P13-PC) Q15. I’m really interested, so because obviously I know from my perspective of how it is, but I’m really interested to hear the health professional’s perspective (P01-L) Q16. I remember the first community of practice meeting everybody was a bit confused [laughs]. I think we were all sort of there, because it was right at the beginning, we had a blank piece of paper really. We knew we had this document; we knew we had this document with good research in it and we knew we wanted to get some of it out into practice, but we didn’t know how to get from A to B. We didn’t know which research studies in there would be the most important and I think everybody was kind of looking at each other for sort of one person to lead (P06-PNC) Q17. One of the patient members told me she felt like she was in the community of practice, I think it was maybe the fourth one, she said, ‘I feel like I’ve had my training wheels on and I’ve been learning to drive for the past three meetings, but now my training wheels are off and all of a sudden I’ve worked out how to do it’. So she said it was a kind of lightbulb moment of, ‘Oh, I get it now, I understand clinical pathways, I understand how research can fit into them, I now understand how to, how I can comment on that and maybe make a difference’ (P06-PNC) Q18. I would say the outcomes of that meeting steered the direction for the next community of practice meeting. As we’ve gone on, we then realised that it would be effective to have a patient steering group run alongside so, it’s given oversight that’s challenged and questioned what the priorities are for the local area and that’s helped formulate the agendas going forwards (P15-PNC) Q19. It’s (the CoP agenda) been driven by a combination of the members of the community of practice dictating from the outcomes of the work but also then influences from patients and influence from wider work within the unit (P15-PNC) Obs4. (meeting 2 face to face) all public contributors appear confident in meetings, for example, all took turns to speak, raised hands, asked questions, interacted with others in group work Obs5. (meeting 3 face to face) Session this morning slightly uncomfortable – tasks from the last meeting were distributed to clinicians/team shared on the screen and had not been completed ? due to time (too busy)
	CoP membership	Recipients Context	Q20. Having commissioners there and I know the GP was there…hearing them say what’s important to them might actually be more powerful than us from a service delivery point of view saying, ‘Well this is what we can do with the money that you give us’ and having the patient say, ‘Well actually this is what’s important to us’ and focus on that and how we then deliver services around that. So I think that’s where [the CoP], it brings those key players in and it’s not just a sort of provider, fund-holder decision all the time (P11-PC) Q21. Having those two sides to my role has been really, I’m really fortunate because I’ve got access to the research and the researchers, and I’ve got time allotted to that, as well as the clinical networks that you need, and the influential role that I have being a consultant physio. So I get to go to meetings such as STP meetings where I’m meeting commissioners and meeting people….In lots of ways I’d got all the dominoes lined up, I’d just got to link them all together. So it was kind of knowing the area and knowing the people in the area in order to be able to influence them (P07-PC) Q22. I have had user experience before in terms of patient participant groups from GP practices we’ve had to go and speak to and things, but it’s only ever been when we want to make a big change and actually – and I think what Moving Forward has I suppose opened my eyes to is that we shouldn’t just use them when we have to, they should be integrated into whatever we’re doing and it should be that sort of co-production, it shouldn’t again be, ‘Well this is what we’re gonna do because we think it’s the best way’. You know, it should be a joint decision (P11-PC) Q23. I remember hearing quite a lot at the beginning about how amazing it was that patients and the public were there around the table, around the same table with commissioners and NHS partners. But I remember thinking, ‘This shouldn’t be something new, this should be taken for granted, this should be just normal (P06-PNC) Q24. I think the patient group are very educated, very well informed. But I see some patients perhaps at the other end of the spectrum who perhaps aren’t able who articulate their problems very well, are still expecting someone can fix them. And there are still those barriers there, so around the table although we’ve got a patient group, a patient voice, it’s fantastic, I’m not sure it represents the spectrum of patients that we deal with. And that’s not a criticism because how do you invite them when they don’t know it’s really, really difficult isn’t it. Just as being mindful that in our patch we’ve got people who don’t speak English, lots of people. And very, very many and there are patches where we’ve got lots of deprivation. And I think my concern, and I’ve voiced it a couple of times, is social deprivation and inequalities (P07-PC) Obs5. (meeting 5 online) Public contributors appear well versed in research. Good understanding of research process and methods. One public contributor made reference to the fact their neighbour ‘wouldn’t understand all of this’
Culture and relationship building	Relationships and opportunities	Innovation Recipients Context	Q25. What I found really interesting is having the commissioners there and being able to influence by showing them this is what we’re capable of, this is where we’re at in terms of influencing best practice, you know, best care for patients (P05-PC) Q26. It’s trying to make them [commissioners] understand what is best practice and what should we be doing and getting them on-board and actually them saying, ‘Well here’s your money, you deliver it how you see fit’… and what’s best for the patient, how we as providers see fit to deliver it as opposed to them dictating, ‘Here’s your money, but we also want you to do X, Y and Z’ (P11-PC) Q27. Finally, we have something that we can take to commissioners or to whoever. You know what, if you’d like evidence, yes, no problem I can give you the evidence, here’s the booklet (P13-PC) Q28. I think the very fact that they’ve got that document now, and they’ve got the evidence that backs it, it’s much easier to go and sell that to a service if you’ve got endorsement from all areas, the researchers, the commissioners, the patients (P07-PC) Obs6. (meeting 3 face to face) The commissioner is always ‘popular’ at break times. Clinical staff appear to gravitate towards the commissioner. Several questions this morning directed towards the commissioner
	Power balance	Recipients Facilitation	Q29. I think with hindsight that’s probably a very good thing to empower them. They went into those meetings knowledgeable, probably more knowledgeable than the clinicians and others that were hearing it for the first time (P03-PNC) Q30. So they’ve gone through it, they knew what Moving Forward was, they’ve reviewed the evidence, they’d thought about it where they thought the priorities were and they went into that meeting with everyone saying you’ve got an equal voice, but they were prepped with it. Whereas all the other clinicians and the commissioners and the managers just came in blind. You know, they’d been sent a copy of it, but we hadn’t done that prep work. We expected them as professionals to be able to read it (P03-PNC) Q31. I feel privileged and honoured to be in the inner sanctum and have a seat at the top table, little old me, at a relatively low-level position, i.e. a service user (P02-L) Q32. The challenges first of all were to gain the confidence to accept that you are on an equal footing and that what you have to say is as important (P08-L) Q33. I think what’s been special about erm our community of practice moving forward is that it doesn’t feel like a normal meeting…it doesn’t feel like a meeting of stakeholders coming together to discuss something, put a plan in place and then go home again. It feels – everyone looks forward to it. Erm because we’re using different creative methods to get us thinking, we’re not just staring at a PowerPoint and making notes or you know, brainstorming on a piece of paper, we are – xxx getting everybody up and talking to each other. You know, talking about –using personal things to get people having that conversation to really start to breakdown those boundaries erm and just sort of thinking a little bit differently (P06-PNC) Q34. Everybody is given a little section throughout all the different community of practice meetings. We’ve heard from the commissioners, we’ve heard from the clinicians, we’ve heard from the patients, we’ve heard from knowledge brokers, we’ve heard from project managers, so it’s not just the project leads that have speared it, it’s everybody together and everybody has had the chance to take the floor and talk about their own context and how they can work together to drive the project forward (P06-PNC) Q35. Nobody is being talked down to or talked at, whoever it is that’s talking. So you know, the coaching and mentoring bit is quite soft and subliminal, but it’s there. Erm lots of delegation going on, it’s not you know, ‘You will do this, you will do that’, it’s you know, ‘How can we do it? Who would like to take this on?’ and people are cajoled in a sort of roundabout sort of way to putting their hand up (P01-L) Q36. So, there’s been a lot of prep work. In terms of the meetings, there’d been a lot of thought about who would go with whom. So we knew we’d break out into groups… we made the decision to have mixed groups when we broke up into groups. So it would be a patient, a commissioner, a GP, a clinician, and a facilitator from the IAU, facilitators had been prepped – this is the tool going to use and how to get there (P03-PNC) Q37. In putting the agendas together, considering what areas we want to cover, understanding and thinking about how PPIE fits in with that and how the patient voice can be brought through the community of practice so getting that balance right that we’re not just producing an agenda that fits the needs of the clinicians that are coming to make sure that patients are fully involved (P15-PNC) Q38. We always get really positive feedback from xxx and xxx and xx to say you know, ‘Thanks for grounding us again and keeping us focused on what we are actually trying to achieve here and what direction we’re going’. Because as lay people, if we don’t understand what’s going on then for me it’s like well what’s the point? (P10-L) Q39. We had this discussion about ‘Who’s gonna chair?’ and of course xxx and I never even dreamed that it’d be us. It was like, ‘Which one of you professionals are gonna do it?’ and xxx just threw into the ring, ‘Why don’t we have someone who’s not you know, not involved in the research day to day?’ and she said, ‘What about you’? (P10-L) Q40.It’s not very often that they [clinicians] have to engage a patient in that capacity. They're usually only have to give instructions, I know that sounds harsh but that’s what they're there for, to give instructions. So, to be on an equal standing, it took a little bit of time for that to develop (P08-L) Q41.You could see the surprise, you could see the almost discomfort I think at times that you know we don’t work with, we don’t see patients in this capacity, I’m normally treating you I never ask you what you think and you know the two way working they’re just not used to, but interestingly although we could see that emotion come through in the meeting and nobody backed out of the community of practice so, we didn’t get clinicians walking away going, ‘I can’t work in this environment’ so, it was a challenge that they accepted (P15-PNC) Q42. It gave the patients a platform, it empowered them to challenge and then from that challenge they could then coproduce going forward it almost gave them permission then to work alongside the clinician (P15-PNC) Q43. I think they’ve [lay contributors] given a reality check, you know, when I’ve been in meetings, they’ve given a reality check. But they’ve also given an aspirational view, as a patient, ‘this is what I want’. But I think maybe, it may have also given them a reality check as well, ‘okay we want this but there are some barriers’ (P07-PC) Obs7. (meeting 5 online) feedback following group work—Public contributor used language indicative of power imbalance ‘you’re the experts’, yet appear very confident feeding back to group and expressing opinions Obs8. (meeting 2 face to face) IAU staff regularly checking that public contributors ‘are ok’. There is a dedicated person (knowledge broker) to support public contributors who has good relationship with them. No one appears to ‘check’ that clinical staff are ok—? assumed Obs9. (meeting 3 face to face) Social icebreakers to each session are excellent…..great energy, welcoming, feels like the meeting starts by levelling the playing field by talking about something that is accessible to everyone…..social space for lunch, coffee, cake appears an important focal point of the morning. Good integration at these times—Public contributors don’t appear to ‘stick together’ because they know each other
	Enabling co-production	Recipients Facilitation	Q44. The beauty of it was that we were all in the same boat together and that discussion between us all led to a solid plan (P06-PNC) Q45. That has been the big aim that everything is coproduced, so, no one is right or wrong but we’re all together, I think we had an idea of that, but we weren’t sure just how it would work. Whether our patient voices or public voices would be heard and whether they would be valued because obviously it’s a learning curve for some of the professionals (P08-L) Q46. At first, we were kind of like rabbits in the headlights, you know when we met each other at lunchtime, cause we were sort of, are you getting this, you know do you understand and I think just the fact that we all felt the same, was enough support to know that we weren’t going wrong anywhere, we were all just not used to this sort of communication if you like (P08-L) Q47. She’s [lay contributor] definitely found her voice and she has after every community of practice recently has emailed to say how much she’s enjoyed it and how much she feels like she’s starting to make a difference, whereas at the beginning she kept questioning why she was there and what difference, why would she make a difference, why would her voice be important (P06-PNC) Q48. Once you understand what mobilisation means and you know, all the different things that they use, once you understand what they mean then you can just use our normal day to day language – they would understand me as a patient (P01-L) Q49. I saw the GP and commissioner’s mind change because of what the patient said, and I also saw the patient’s mind change. And that to me was collaborative practice (P03-PNC) Obs10. (meeting 2 face to face) All activities appear very social, well planned, involve moving/going outside/require different skills. This facilitates contribution from everyone. Questioning from patients this morning prompted reflection from clinical lead
Responding to external context		Context Facilitation	Q50. Now (during covid) it’s easier because with the message facility on here I can just put a message on and say, ‘I don’t understand what you mean (P10-L) Q51. Although Covid, you wouldn’t wish it on anybody would you or this situation or pandemic at all, it kind of gave us an opportunity in one sense if you look at the silver lining to step back from that clinical work and concentrate on what is the current evidence out there? What should we be doing and how can we now translate that into our clinical practice? (P11-PC) Q52. I saw it as an opportunity to make those changes, to have the time to implement that we’d never had before (P05-PC) Q53. The first lockdown what we did, because we didn’t have as many patients coming through the doors, we’ve done a lot of pathway work and looking at best practice…. It probably would’ve taken us years before and now I think we’ve already done the research part; we’ve done the looking at the evidence (in the CoP) (P11-PC) Q54. I think Covid has changed the priorities, but I think our community of practice have helped to stay connected with important issues (P03-PNC) Obs11. Tension noted re discussion ‘should we change priorities in light of covid’ (overlap with negotiating knowledge) – local clinical guidance and shared best practice on remote consultations. Obvious divide and mixed opinions between members who felt that the Moving Forward priorities should be revisited and clinicians, whom for some, there was a strong sense of ‘we can’t cope with anything else right now’. Members appeared frustrated at the thought of ‘starting from scratch’. IAU staff helped facilitate compromise for next steps Obs12. This was the first online CoP post covid – a very emotional meeting indicative of a ‘safe space’ for all to share experiences. Bereavement of friends and colleagues discussed. This ‘emotional offload’ appeared important to let members know they were supported (lots of positive messages of support in the chat box) and to set the tone and agenda for future sessions. Everyone made mistakes together regarding technology etc. using MS Teams – a strong sense of learning together

*PC* professional clinical, *PNC* professional non-clinical, *L* lay member

### Public contributor involvement

For the purposes of this paper, the term public contributor is used to describe patient and public involvement in this research study. The term ‘lay member’ is used to describe patient and public members of the CoP.

Public contributor involvement, reported in line with the GRIPP2 checklist, [43], included:

- Study development—advising on study design through a dedicated co-applicant role on the research grant funding

- Interpretation of study findings and co-production of recommendations and study illustration—via dedicated meetings and subsequent communication with two members of the Lay INvolvement in knowledge mobilisation (LINK) Group (which supports meaningful engagement in the implementation of research evidence into practice) from Keele University and one member of the Research User Group [44]

- KM activities—including collaborative working with an illustrator to develop a visual representation of study findings suitable for a wide audience, and contribution to study write-up as a co-author (KF)

## Results

Four CoP meetings (two face-to-face and two online) and one planning meeting were observed (duration 65–200 min). Fifteen multi-disciplinary stakeholders from the CoP participated in semi-structured interviews: five lay members, six clinical professionals (e.g. Advanced Physiotherapy Practitioner, Service Lead), and four non-clinical professionals (e.g. managerial, knowledge broker, commissioner). Four men and eleven women were interviewed (duration 29 to 60 min). Five individuals did not respond to the study invitation (four clinical and one non-clinical); however, theoretical saturation was considered to be achieved by the 12th interview.

In exploring the process of KM in a CoP for implementing best evidence in musculoskeletal care, four overarching themes were identified: Identifying and interpreting knowledge; practical implementation of a CoP; culture and relationship building; and responding to the external context. The four overarching themes, subsequent sub-themes, relationship to i-PARIHS and supporting quotes are presented in Table 1 and discussed below.

### Identifying and interpreting knowledge

*‘This is an opportunity to really fully embrace the evidence’ *(P07-PC).

#### Types of knowledge

Several different types and formats of knowledge were valued, used, and amalgamated to inform decision-making within the CoP, relating to the implementation of best musculoskeletal care [Q1,Obs1]. CoP members integrated research knowledge from Moving Forward (the innovation) with clinical knowledge acquired through training accreditation, practice-based experiential knowledge, stories (lay and professional), patient and public knowledge, and audit data.

#### Knowledge creation and use

Despite the demand for best evidence in clinical services, the Moving Forward Themed Review was deemed ‘too dense’ and complex by both clinical and lay members [Q2]. Therefore, an early sub-project undertaken by lay members alongside the LINK group was the development of a public, ‘easy read’ version of Moving Forward [45]. Furthermore, in CoP meetings, research knowledge from Moving Forward was explored in relation to other knowledge types as well as individual and local context. For example, decision-making within the CoP for osteoarthritis (OA) services was influenced by local audit data, contextual factors such as geographical variation, clinical leadership, resource (financial, staff, venue), and knowing the ‘key players’ for influencing change.

The social context of the CoP involved creative methods and discussion from multi-stakeholder perspectives which engaged all members (recipients), eliminated boundaries, and instilled confidence in communicating and considering new ideas. ‘Group think’ transformed evidence and generated new, socialised knowledge for solving implementation challenges in practice [Q3].

#### Negotiating knowledge

Tension was noted regarding the academic agenda to acquire and use research knowledge from Moving Forward and the clinical agenda to respond to clinical pressures in a timely manner [Q4]. Furthermore, participants described instances whereby the research requirements were incongruent or at odds with the offer that could be delivered in clinical practice[Q5,Obs2], therefore knowledge was negotiated with facilitation within the CoP.

Lay members described feeling that they lacked knowledge about the project in the early stages of the CoP, furthermore, they assumed that the clinical knowledge of healthcare professionals (HCPs) was ‘preferred’ (by other CoP members), more useful, and subsequently valued more than their own experiential knowledge. Lay members also assumed that HCPs had a shared professional, tacit knowledge (e.g. they ‘know’ the same thing), to include knowledge from Moving Forward, which was not always the case [Q6]. In addition, lay members expressed how their ‘lay status’ reduced the perceived expectations of others regarding their knowledge contribution [Q7].

Patient and public knowledge was difficult to disregard by other stakeholders in CoP meetings. However, this appeared problematic when group interactions focused upon the funding of services. In one instance, knowledge relating to lived experience of a condition was insufficient in ‘persuading’ the group to change practice due to restrictions imposed by funding. Lay members did not have detailed knowledge or understanding of the different types of 'service and system' knowledge that required amalgamating and negotiating for implementation.

### Practical implementation of a CoP

‘*You need a strong support team’ *(P15-PNC).

*‘The way it was organised was fantastic you know, in the diversity of the people that were there and also how they went through things. And then having clear outcomes and things to work on’* (P05-PC).

#### Infrastructure and support

The internal context and successful operationalisation of the CoP appeared to be optimised by support (or facilitation) from the IAU team. This included organisation (such as regular pre-CoP planning meetings), project management, lay member support, financial oversight, communications input, and a project steering group (led by lay members) [Q8-10, Obs3].

Facilitation of the CoP was led by the IAU team who utilised a range of interactive methods i.e., to engage members. This enabled the CoP agenda to be driven by the group, whilst being responsive to contextual demands. A sense of enabling leadership was noted as members were encouraged to take ownership of components of the project [Q11].

#### Clarity of aims and purpose

From the recipients’ perspective, clinicians viewed their involvement in the CoP as part of their job or professional role, described by one participant as their ‘raison d’etre’. In contrast, lay members expressed uncertainty as to their role within the CoP and whether this related to their experience of a particular condition, use of healthcare services, or experience of involvement in research (Obs4).

Participants did not perceive the goals and purpose of the CoP to be explicit from the outset. Tension was noted in the data regarding the iterative nature of the approach, meaning that members were unable to fully prepare for meetings or understand the ‘direction of travel’. This created discomfort for some lay members but there was no evidence to suggest that clinicians felt the same.

Despite many participants describing initial uncertainty regarding the goals or purpose of the CoP, the hope of addressing individual motivators was a reported reason for attending. For example, clinical participants wanted to keep abreast of best evidence, whilst commissioning participants sought to avoid duplication of work by collaborating with a team of ‘credible expertise’ [Q12-14]. In contrast, lay members described their sense of enjoyment and interest in the field and the desire to learn more [Q15].

The CoP process was described by one participant as a ‘learning journey’ together, starting with a ‘blank piece of paper’ whereby the outcomes of one meeting would steer the direction of subsequent meetings [Q16-17].

Clarity of the CoP goals and purpose evolved over time. Related to this, one participant described how a task-orientated meeting style, where the purpose did not appear to be clear, was ineffective (Obs5). Following reflection and with steering group oversight, an active, more collaborative facilitation model was adopted which enabled members to identify and navigate the group goals and process, and the new knowledge created (e.g. regarding clinical priorities and which research evidence to integrate within services) [Q18-19].

#### CoP membership

Whilst the CoP goals and purpose appeared fluid and flexible, the membership remained consistent throughout the project duration. A diverse, multi-disciplinary membership was an important feature of the CoP context and culture that enabled an understanding of others’ professional drivers, reflection on CoP activities from different perspectives, equal sharing of decision-making, and the development of co-produced outputs [Q20]. Furthermore, the inclusion of stakeholders with boundary spanning roles who were able to influence decision-making was important for taking actions from the CoP into clinical practice [Q21].

Participants had mixed views as to whether the CoP membership was suitable for enabling changes in practice. Some expressed the view that more senior commissioning partners were needed along with clinical leadership, whilst others described the benefits of including more junior staff who were involved in the day-to-day running of clinical services.

Lay involvement in implementation was novel or unique for some participants, yet the value in shaping the project was acknowledged. This prompted reflection and action by clinicians to address patient and public involvement within the clinical setting [Q22].

One participant expressed the view that patient and public involvement should be commonplace throughout healthcare service delivery and implementation [Q23]. Despite this, several clinical participants described concerns relating to the representativeness of the lay members, whilst acknowledging the challenges associated with achieving a broad, representative patient and public group [Q24, Obs5].

### Culture and relationship building

‘*I saw the GP and commissioner’s mind change because of what the patient said, and I also saw the patient’s mind change. And that to me was collaborative practice*’ (P03-PNC).

#### Relationships and opportunities

Participants reported the benefits of developing relationships with other stakeholders during the CoP process. Clinical participants valued building relationships with academics to enhance their understanding of research, whereas lay members valued the opportunity to better understand a commissioning role. Several participants held the perception that the physiotherapy profession was under-valued by commissioners. Clinical participants perceived commissioners to ‘have the power’ to make changes to service delivery and saw the CoP as an opportunity to influence their relationships and decision-making (supported by evidence), and to demonstrate the capabilities of the profession [Q25-28, Obs6].

The CoP provided an opportunity for multiple stakeholders (recipients) to consider best evidence (innovation) in partnership rather than working ‘in silos’. Silo working was described by one clinical participant as not having all the key decision makers (e.g. an operational lead, manager, commissioner, and business lead) in the same room and having ‘separate conversations’ regarding service delivery.

#### Power balance

A commonly held assumption by lay members (recipients) was that clinicians had knowledge and understanding of Moving Forward, despite only one clinical participant describing prior knowledge. Lay members described the challenge of ‘accepting’ that they were on an ‘equal footing’ with others and that their contribution was important (Obs7). Additional support (facilitation) was provided by the IAU team to lay members (in the form of pre-CoP meetings) to facilitate understanding of Moving Forward and reinforce their potential role within the group. Consequently, findings suggest that at the outset, lay members had *more* knowledge about Moving Forward than clinicians [Q29-30, Obs8]. However, a lack of confidence in challenging clinicians and group discussion in the early meetings was described but not observed, with language used (interviews and noted during observations), suggestive of a power imbalance [Q31-32, Obs 7–9].

Developing an equal standing between CoP members empowered lay members to challenge discussion and influence decision-making, which enabled a shared language and knowledge to develop, facilitating co-production. Lay members valued speaking to others in a social capacity over lunch and features of the facilitation approach that provided an equitable platform included: a neutral hospitality venue (pre-covid-19), the relaxed, welcoming, and collaborative style of the facilitator, ‘fun’ and informal ice breakers, and the use of creative methods which offered an equal weighting to all stakeholders regardless of background [Q33-35, Obs9]. Prior consideration and planning of meeting agendas, structure (including a variety of speakers), and activities (such as member selection for small group work) by the IAU team was deemed beneficial [Q36-37].

Completing a group task (e.g. developing the public version of Moving Forward) and receiving feedback were important for lay members in establishing equity and harnessing the direction of travel by providing purpose and common ground [Q38]. This was enhanced for two lay members who chaired the CoP steering group meetings [Q39].

Initially, it appeared the role of the lay member was not commensurate with contributing clinical or experiential knowledge to interactions with clinicians. An ‘unusual’ shift in clinician-lay member dynamic was described. Interactions involved lay members challenging or questioning clinicians in the CoP meeting contrasted with clinical consultations where typically the clinician may be seen to hold power and translate knowledge to the lay member (patient) [Q40-42].

Lay members appeared more aware and more comfortable with this altered dynamic than clinicians. One lay member described the process as a ‘learning curve’ for professionals. In contrast, a clinical participant explained how they hoped that attendance at the CoP would enable lay members to glean insight into the challenges faced by service leads and commissioners [Q43].

#### Enabling co-production

The CoP culture along with facilitation of activities enabled knowledge sharing, generation of new solutions to complex problems, and co-production of decisions, action plans and outputs [Q44, Obs10]. Trust and respect were important features of the CoP context which enabled members to feel empowered, safe to respectfully challenge ideas and to develop positive relationships. This developed, over time, through engaging in a social capacity, discussing knowledge and implementation from different perspectives, and individual and group reflection [Q45].

Factors affecting member participation and subsequent co-production in the CoP included a lack of research knowledge (academic skills) for one clinician and insufficient knowledge of service design and delivery for lay members. The language used within the Moving Forward themed review and in the CoP, took time for lay members to feel comfortable with. This affected the confidence of lay members and *if* and *how* they contributed to discussions and decision-making [Q46-48].

Participants described several instances whereby, viewpoints of CoP members changed because of group discussion with a variety of stakeholders, allowing the group to come to a shared decision [Q49]. The central meeting venue (pre-covid-19) reportedly enabled co-production by allowing clinicians to step outside of their working environment and discuss issues freely without fear of judgement.

### Responding to external context

‘*I think Covid has changed the priorities, but I think our community of practice have helped to stay connected with important issues*’ (P03-PNC).

The main external contextual factor that affected the health system and all CoP members was the COVID-19 pandemic. COVID-19 impacted upon the process and decision-making of the CoP in several ways.

The group initially agreed to suspend meetings whilst considering changes in practice. Following this, the IAU team organised the transition and facilitated virtual CoP meetings. The positive and negative impacts of the digital environment on KM and the power balance within the CoP was described. Whilst some participants felt that virtual meetings were more tiring and stifled enthusiasm, one lay member suggested that virtual meetings enhanced equity by enabling them to ask questions using the chat function if they lacked confidence [Q50]. For many, virtual meetings were convenient, however, one lay member was unable to attend virtually, thus highlighting the potential for digital exclusion.

The CoP re-visited their initial priorities and discussed whether these remained relevant given the change in context (Obs11). For some participants, the potential of changing tack appeared problematic (created chaos) and unsettling. This was mainly because clinicians were experiencing unprecedented amounts of change in practice but also because some lay members felt strongly that the work undertaken to date should not be side-lined to start a new work package. As a result, the group continued with the agreed priorities but considered their delivery in light of covid-19. Participants described how the CoP provided members with the space to make sense of new knowledge, discuss with others and consider different strategies for implementation [Q51].

Several ways in which relationships and opportunities were affected by the covid-19 pandemic were reported. Some participants described colleagues’ fear and the need for stability in times of uncertainty. Others described how the change in context had freed up action time, reduced barriers to organisational change, and provided ‘permission’ for service change [Q52].

Clinical participants described how the CoP had supported and enabled them to see a range of opportunities to plan service re-design and enhance service delivery that would have taken years to implement in pre-covid-19 circumstances [Q53]. The CoP continued to support members during the pandemic, enabling the group’s aims and purpose to flex and adapt to changes in clinical context [Q54, Obs12].

## Discussion

**Fig. 1:**
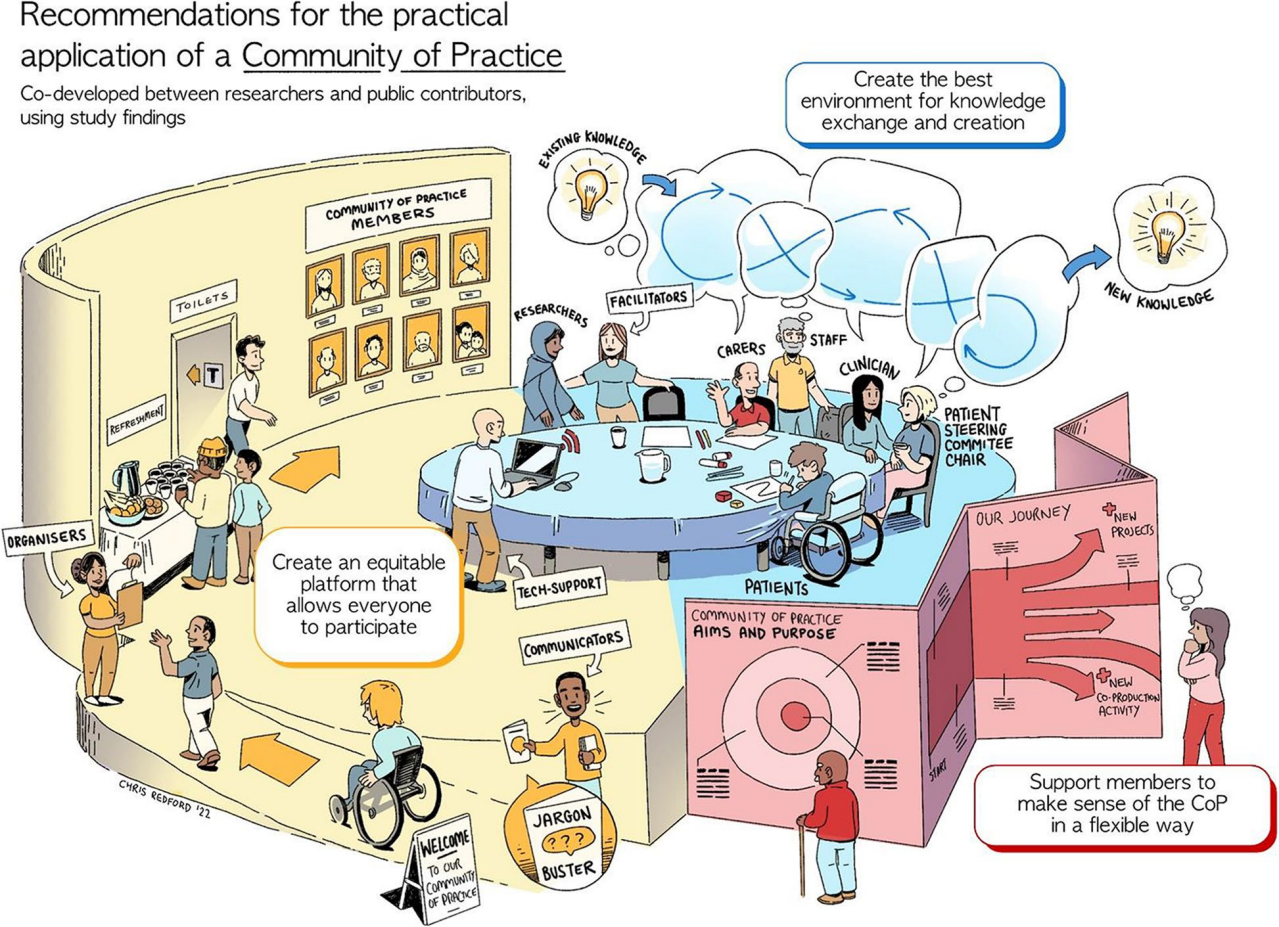
Recommendations for the practical application of a Community of Practice

This qualitative study has utilised the i-PARIHS framework to explore the process of KM in the context of a CoP to implement evidence-based interventions in musculoskeletal care. Creating the best environment for knowledge exchange and creation, creating an equitable platform that allows everyone to participate, and supporting members to navigate the CoP in a flexible way were important features of the CoP context. Findings yield a greater understanding of the ways in which clinical, commissioning, lay and academic stakeholders (or recipients) connect, engage, learn, and support each other in a CoP and how a supportive infrastructure facilitated this. This study has identified how a CoP with diverse membership can promote partnership working at the intersection between knowledge and practice when addressing the uptake of an NIHR themed review. We highlight the importance of support and facilitation, particularly the role of IAU in establishing, and maintaining the CoP to develop a culture conducive to relationship building and co-production. The CoP facilitated the creation of new, socially constructed knowledge, which took a range of stakeholder perspectives into account. Novel findings relate to the role and involvement of lay members in an implementation CoP, and the iterative, flexible nature of CoP aims and purpose according to context. Recommendations for the practical application of a CoP to optimise KM are presented in Table 2, depicted in Fig. 1, and discussed below considering supporting literature, relevant theory, and public contributor involvement.

**Table 2:** Recommendations for optimising the process of knowledge mobilisation in Communities of Practice

Aims	Recommendations
To create the best environment for knowledge exchange and creation To create an equitable platform that allows everyone to participate To support members to navigate (make sense of) the CoP in a flexible way	*Pre-Community of Practice* ••••••••••••••Establish an infrastructure to provide administrative and wider support to the CoP group and to facilitate meetings. To include a team with a broad skill set to assist with securing necessary funding, organisation (of people and place), technical and digital expertise, project management, regular communication (announcements, news sharing) ••••••••••••••Include a broad, diverse range of stakeholders, including lay contributors (e.g. patients, carers, service users and the public) to provide a breadth and depth of perspectives ••••••••••••••Consider the physical environment (e.g. a neutral meeting venue, provide refreshments) to ensure members feel relaxed and optimise engagement ••••••••••••••Provide the opportunity for pre-CoP support for all members (e.g. preparation work may be required at the beginning to familiarise members with broader aspects of research or implementation (including terminology/language), to provide clarity regarding their potential role within the group, and in understanding potential aims and purpose of the group ••••••••••••••Offer more formal roles (e.g. chair to steering group meetings) to patient and public members *Ongoing* ••••••••••••••Consider the agenda and structure of meetings, including member selection for small group work and co-production activities that give members a sense of ownership over the project ••••••••••••••Where possible, by discussion, identify and agree the aims and purpose of the CoP at the outset. If this is not possible, explicitly acknowledge the iterative nature of the CoP process and that the group aims, and purpose will evolve over time ••••••••••••••Consider the varied skill set, knowledge base and expertise that all members offer and recognise that more support may be needed to identify how, when and where patient and public knowledge is applicable to implementation discussions, decisions, and action ••••••••••••••Facilitate relationship building, the integration of stakeholders, and co-production (e.g. by using creative and social strategies for engagement, enhancing partnership working, creating a culture of trust, and empowering members to contribute effectively). Group tasks can harness direction of travel by providing purpose and common ground ••••••••••••••Utilise collaborative facilitation to enable group members to identify and navigate group goals and purpose ••••••••••••••Allow the CoP ‘direction of travel’ to accommodate change and to be flexible and responsive according to context

Variability in the structures and interpretations of CoPs makes it challenging to operationalise the approach in healthcare practice [29]. Self-organisation and self-regulation are reported features of CoPs whereby it has been suggested that advanced CoPs, which can transform care, thrive and survive without individual or organisational support [28]. In contrast, our results are consistent with the i-PARIHS theory and recent work highlighting the importance of support and dedicated facilitation in CoPs seeking to implement research evidence into clinical practice [10].

This study contributes to the CoP literature by identifying the importance of resource and a supportive infrastructure via a multi-disciplinary team of people. Using the lens of i-PARIHS, the IAU played a facilitatory role throughout the process of the CoP which was central to its functioning by creating an environment (internal context) for knowledge creation and exchange. This is consistent with our previous work detailing the ways in which resource and infrastructure can support the workload of implementation [32]. This study illustrates how the broad skillset within the IAU was pivotal in firstly, securing funding to establish a CoP, and secondly, creating the culture (internal context) for the group (recipients) to collaborate and flexibly co-produce relevant implementation solutions.

The challenges of transferring research knowledge from an innovation and achieving successful implementation in healthcare are widely reported [11, 46]. This study illustrates how different types of knowledge were negotiated from a range of stakeholder experiences and how this led to the co-production of new knowledge and collective ownership of decisions, applicable to local context. By facilitating interactions between colleagues, opinion leaders, and patients, CoPs offer the opportunity to mobilise and transform both tacit and explicit knowledge to be useful in practice [47, 48].

One of the intended functions of CoPs is to bring together stakeholders who may not otherwise work together [28]. Whilst implementation activities are typically led by HCPs, and ambiguity exists surrounding both the role of patients and the public in implementation [32, 34] and in CoPs [17], this study illustrates how a CoP offered an appropriate forum for integrating the voice and knowledge of all recipients, including patients and the public, into healthcare implementation, in line with NHS policy [49, 50]. However, an altered dynamic of more equal power sharing was seen between HCPs and lay members working together in a CoP when compared to either a research project or a clinical consultation. This is consistent with literature describing how patients' experiential knowledge is perceived to be of lesser value than clinical knowledge (by both HCPs and patients) in clinical consultations [51]. Current CoP literature identifies a lack of clarity in how to best manage power dynamics in CoPs [29] and the importance of support in ensuring that all members have an equal opportunity to contribute [52]. Our study illustrates the importance of facilitation and careful planning of CoP structure to mitigate issues relating to power dynamics.

Our findings demonstrate that more support may be needed to identify how, when and where patient and public knowledge is applicable to implementation discussions, decisions, and action. Merely inviting lay members to the table is insufficient and has the potential to reinforce ‘tick box’ mentality as described in literature regarding PPIE in research [53]. With appropriate support (in understanding their role and the terminology associated with implementation, to negotiate meaning within the group) and using carefully considered facilitatory approaches, patients and the public can challenge both HCPs and the balance of power to contribute to decision-making in healthcare implementation. This is consistent with our earlier work[32], illustrating the overlap between the facilitation and recipient constructs of i-PARIHS when considering the role of the public in implementation.

Whilst the CoP reported in this study came together with an implicit overarching purpose, with funding from the CSP, to implement the best evidence from the NIHR Moving Forward Themed Review, it was apparent from the data that members were uncertain of the group’s aims and purpose. Whilst uncertainty regarding the CoP aims and purpose caused some initial discomfort for (mainly lay) members, the iterative approach to the ‘learning journey’ enabled members, overtime, to make sense of the CoP itself, build relationships, co-produce decisions and gain a sense of trust and ownership over the project. CoP members needed to understand and make sense of the CoP to decide whether they want to take part and subsequently take action to make changes in practice.

The fluid and flexible approach towards the group aims and purpose was beneficial in enabling co-production and was also of value when responding to the context of covid-19. Current literature has alluded to the negative impact that covid-19 had on CoPs (described as a ‘drawback’) [10]. In contrast, our study identified the CoP afforded opportunities to mobilise knowledge and problem-solve COVID-related issues arising in clinical practice. The creation of a safe space and trusted relationships enabled the group to discuss their original priorities. This was however set within the context of CoP members who were aligned in their vision to improve the quality of musculoskeletal care, which remained a priority in the local healthcare system.

A key strength to this study was the inclusion of lay, clinical, commissioning, and academic stakeholders to glean insight into a CoP from a broad range of perspectives. Furthermore, to ensure the trustworthiness of this research, steps taken include double coding, along with the use of illustrative quotes and triangulating two data sources to support interpretations [54]. Conducting participant observations online, a challenge presented by covid-19, had the potential to negatively impact on the richness of data collection and analysis. However, some participants did report feeling more confident to participate online and we believe that the combination of online and in-person observational data, supplemented by interview data, gleaned valuable insights, and presented an authentic account of the CoP [55–57]. Likewise, an obvious drawback to the study is the Hawthorne effect (behaviour change as a result of being observed), however, similar to Mulhall (2002) we believe that it would be challenging for CoP members to maintain different behaviour over a sustained period of time, especially considering that CoP members were busy and focused during meetings [37].

A further strength to this study was the use of theory which enabled us to ask questions of our analysis in relation to the constructs of i-PARIHS. This advanced our interpretation of the data and led to our development of themes. This was also important for guiding our recommendations by enabling us to move beyond our initial insights to understand the broader significance and applicability of the phenomena under study. We used the lens of i-PARIHS due to its applicability as a conceptual framework to explore the actors and actions of KM yet recognise there is overlap with other theories which may have led to different interpretations.

Public contributors have influenced several important aspects of the study including the recommendations and study illustration to support meaningful KM. Feedback was provided by the researcher to public contributors to illustrate the impact of their input (supp material 3). The positive impact of public contributors to this study may be attributed to the fact that they had prior knowledge and experience of CoP methodology, KM, and/or implementation. Established relationships and supported ways of working facilitated high-quality involvement. We acknowledge the representativeness of public contributors as a limitation of this involvement and recognise the need for public contributors to represent the voices of diverse communities that CoPs seek to serve.

The researcher (LS) works within the IAU, however, steps were taken to mitigate any impact of this on the data and to help ensure reflexivity. For example, LS was not involved in the CoP organisation or delivery, and a reflexive audit trail from data collection, analysis and interpretation was recorded and discussed regularly with the study team. KF and ZP were not involved with the CoP. Their input focused on data analysis, recommendation development and KM. This study was grounded within a single implementation project which has the potential to limit the transferability of findings. However, several participants described challenges encountered within the CoP and we believe the findings and recommendations are transferable to others considering the CoP approach in other contexts.

The roles of the study team within the CoP were considered reflexively during data collection and analysis. LS and ZP were not involved with the CoP organisation or contributing to meetings and therefore held several analysis meetings to discuss data before meeting with the broader study team, including KS who facilitated the CoP meetings.

Many of the principles identified in this study are transferrable to CoPs in other clinical fields, e.g. public involvement and the provision of infrastructure and support, yet there are areas where additional work in preparation may be needed such as CoPs with young people and underserved communities. Many unanswered questions about the role of CoPs in healthcare implementation remain. Whilst this study did not seek to evaluate the effectiveness of the CoP, data relating to impact and effectiveness would have enabled a more in-depth exploration of the role of CoPs in implementation. It may be of value to explore what happens at the end of a CoP or when a CoP transitions, to identify any benefits or unintended consequences to members. Furthermore, evaluation of the costs of CoPs is needed to better understand the value added by the approach. More broadly, research is needed to explore the practical application of online CoPs, engaging seldom heard voices in CoPs, and the role of international CoPs in optimising the uptake of innovations and best practice.

## Conclusion

Using the lens of i-PARIHS, this study explored the use of a CoP as a KM strategy to implement best evidence in musculoskeletal care and has generated recommendations to help establish and run a CoP in health service implementation. This study identified the importance of resource and infrastructure for the set-up and facilitation of a CoP and to support CoP members to develop more effective partnership working. By creating an environment that optimised knowledge creation and exchange, the CoP provided a mechanism for stakeholders to negotiate several types of knowledge and co-produce new knowledge (and solutions) to align with local priorities. A key feature of the CoP context was the provision of an equitable platform for participation for all members. It is important to support members to make sense of the CoP aims and purpose in a flexible way and to respond to contextual changes. CoPs offer a potential solution for many of the challenges faced in implementation, yet further work is needed to evaluate their costs, effectiveness, and pathways to impact.

## Supplementary Information

**Additional file 1.**

## Data Availability

The School of Medicine, Keele University, is committed to sharing access to our anonymised research data derived from our population, consultation, clinical, and RCT cohorts. Researchers wanting to apply for access to data from archived studies hosted by the School of Medicine should first email primarycare.datasharing@keele.ac.uk.

## Abbreviations

CoP: Community of Practice
CSP: Chartered Society of Physiotherapy
IAU: Impact Accelerator Unit
KM: Knowledge Mobilisation
NICE: National Institute for Health and Care Excellence
NIHR: National Institute for Health Research
OA: Osteoarthritis
