# Supplementary material for: Optimising the process of knowledge mobilisation in Communities of Practice: recommendations from a (multi-method) qualitative study
Source: Implement Sci Commun. 2023 Jan 26;4:11. doi: 10.1186/s43058-022-00384-1 (PMC9879236; doi:10.1186/s43058-022-00384-1)
Supplement: Supplementary file 1 — Additional file 1. [file 43058_2022_384_MOESM1_ESM.docx]

**Supplementary material Swaithes et al**

# Supplementary material Box 1. Description of the CoP

| **What was the clinical setting for the CoP?**  The clinical setting for the CoP is one of the largest community Trusts in England serving a population of 1.5 million, over a geography of 2,400square miles and employing approximately 8,500 members of staff. Pre Covid, the musculoskeletal service managed 23,000 referrals per year. |
| --- |
| **How was the CoP established?**  The CoP was designed by a Moving Forward project team of researchers, knowledge mobilisers and practitioners with support from project coordination, patient and public involvement and a knowledge broker. The team applied for funding from the Chartered Society of Physiotherapy (CSP) to explore implementation of best evidence from the Moving Forward report. The team were experienced in the application of CoP theory.  A Moving Forward Steering Group (including PPIE and IAU members) met to oversee the overall objectives of the CoP.  Names of individuals with relevant interests/expertise were identified using the IAUs extensive network and invited to join the CoP. This network extended to healthcare, the University, PPIE, charitable sector. The list was iterative, with interested parties added as the project developed. In addition, people volunteered as the work progressed and they started to hear about the work and its ambitions. |
| **What was the structure of the CoP?**  The number of meetings was determined by the funding envelope awarded by the CSP (n=5). The overall structure of the first CoP was designed by KS and approved by the steering group. KS was the main facilitator of the CoP however all stakeholders had the opportunity to lead components of each meeting. The ongoing structure of the CoP was fluid and flexible. Meeting structure was largely determined by the activity of the previous meetings and the priorities identified. Group work and activities were decided according to the aims of each task, numbers present, expertise required, and whether the meeting was face-to-face or online. The agendas had space for project updates and co-creating solutions to challenges faced. Numbers of attendees at each meeting ranged from 15-25. |
| **What types of activities were undertaken?**  Familiarisation and Prioritisation exercise  The aim of the familiarisation and prioritisation exercise was to understand the priorities of local stakeholders and decide which evidence from Moving Forward to implement locally. This enabled i) discussion on the breadth and depth of evidence within the review for clinical, commissioning, and patient stakeholders, ii) to distill and prioritise key evidence relevant to local service context e.g., to get a top 5 priority list of studies from the 55 included in Moving Forward).  Stakeholders were invited to review the evidence in the Moving Forward document in small groups (n=4-6). The groups were configured to ensure representation from clinical, commissioning, and patient perspectives. Initially they were given time to familiarise themselves with the document and could ask questions of ‘roving evidence experts’ (CoP members who were involved in or had knowledge of the original research studies). They were then asked to prioritise the evidence guided by a Prioritisation Framework. The framework required discussion within the community of practice to decide if each individual study within the document was a clinical priority, a patient priority or a commissioning priority. The framework also gave information as to whether the studies were included in NICE guidance and included any return-on-investment data. The community of practice groups then debated each study and applied the prioritisation framework to it. Through this process we were able to prioritise the studies for implementation within the document. This list gave us a starting point for the clinical pathway’s discussion. The subsequent four community of practice meetings then focused on the priorities identified in this first meeting.  Snapshot of Practice  The aim was to understand ‘what was offered’ in relation to current clinical practice before any implementation activities were undertaken. A snapshot of clinical practice was undertaken through an online survey. The questions were developed with members of the CoP and were designed to assess the existing use of best evidence in musculoskeletal care and any gaps in practice.  Co-creation activities  Co-creation lay at the heart of the CoP. All elements were co-created, for example, the aim and the agenda for each of the CoPs through to activities undertaken within the CoP such as co-creating pathways of care. A pathway for low back pain and for osteoarthritis were developed. Consensus across the CoP was sought for the most appropriate way to display the pathways for ease of understanding.  Example of co-creation– Developing a public version of the Moving Forward Themed Review  The patient and public members of the Community of Practice provided feedback on the research featured in Moving Forward in relation to understanding their care. The patient and public group identified challenges with accessing and using the review as it was deemed complex (64 pages long, including over 50 studies).  As part of the wider Moving Forward project, patient and public representatives worked with IAU Knowledge Broker and Research into Practice Officer for the Chartered Society for Physiotherapy (CSP) and the NIHR Dissemination centre to create a public version of the Moving Forward Themed Review. The aim was to i) co-produce a version that was more accessible for the public and that could answer common questions relating to musculoskeletal health, ii) empower professionals and patients to be able to work together to improve quality of life and manage musculoskeletal conditions using best evidence.  Co-production was a central feature of this work, highlighting an example of working across organisational and professional boundaries to support high quality care. The public version (accessible at https://evidence.nihr.ac.uk/themedreview/moving-forward-a-guide-for-the-public/ utilised succinct plain language to present evidence-based information, diagrams and infographics, further signposting, and patient stories. Draft versions were peer reviewed by patients from general practice, the CSP research team, the NIHR Centre for Engagement and Dissemination public representatives, and the authors of the original themed review.  The public guide was launched nationally in October 2020. It comprised 24 pages (compared to 62 pages in the original review). Upon its launch the public version received 591 page views compared to 75 page views for the original review in the same time period. Work was shared via a successful social media campaign, news stories emailed to CSP members in weekly bulletin and featured in CSP magazine.  Group brainstorming activities  Initially CoP meetings were face to face (pre covid-19). Brain storming, discussing, drawing, and visualising clinical pathways enabled the group to understand the challenges of implementing best evidence from a variety of perspectives. Sessions were facilitated to allow all voices to be heard and all views represented. For example, the use of sticky notes to capture CoP members’ ideas allowed everyone’s opinions to be considered. This method allows participants to hear the challenges from all perspectives.  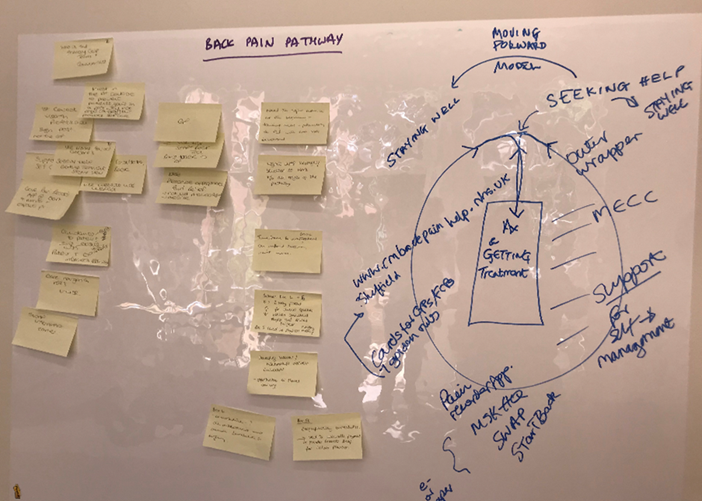  Opportunity for reflection  Interactive methods used within the CoP included the use of cards which required stakeholders to complete part of a sentence to draw out their implementation reflections. For example - “How can we improve implementation…” Responses were themed. Each member chose a theme to explore in more detail in terms of ideas and solutions. Five key themes emerged, these were: communication, expertise, commissioners, co-production, process, and implementation.  In CoP 7, we asked stakeholders to complete the following sentence - ‘Life in musculoskeletal services would be better if…’ Responses included: ‘If I had the chance to meet the FCP; we could simplify a way of sharing information; we had better clinical engagement and representation; we were able to be brave and seize the opportunity; we could have time out; there was protected CPD time’. |

# Supplementary material 2 – Topic Guide example

**Topic Guide: Professional clinical**

Exploring the Moving Forward Community of Practice to implement best evidence for musculoskeletal care

**Background questions related to involvement and current role**

Can you tell me a little bit about your current role?

Tell me about your experience of implementing new models of care/service pathways/ evidence into practice?

*What challenges were you facing prior to CoP with regards to implementing best evidence in practice?*

*What were the clinical priorities in your area?*

**Questions relating to the CoP**

I would like to hear about your expectations of the CoP?

What has been your role/involvement in the CoP?

*Were you clear on your role? what was expected?*

What are your thoughts are about the CoP, how has it helped or hindered your role?

*including how it runs, benefits, and challenges you’ve encountered, how has the CoP agenda aligned to your clinical needs?*

What challenges have you experienced? Why?

How have patients been involved?

*How have they influenced the CoP?*

**Contextual factors affecting the CoP and implementation**

What factors have affected the CoP and Implementation in practice? (positive or negative)

How do you feel about the future adaptation/change of service delivery?

How do you think the CoP will play a role in the future? – *and why*

# Supplementary material 3 – example of public contribution for the study

| We asked… | You said… | We did… |
| --- | --- | --- |
| Is our explanation (including language used) suitable for describing both the public contribution to the research and the public contribution to the community of practice? | - We should acknowledge the different aspects of public contribution - We should define clearly the different aspects of public contribution early in the manuscript - We should be consistent with terminology - We should mention some of the issues regarding public contribution in implementation in the manuscript discussion | - Defined public contributor involvement in the methods section - Reflected on public contribution in implementation in the discussion |
| Is the wording of our recommendations accessible and understandable? Could we present these differently? | - We should make recommendations more specific and add more detail for clarity - We should add more detail regarding the ‘environment’ - We should re-configure recommendations to reflect the recommendations that apply before the first CoP and those that apply throughout the process - We should consider the audience/end user of the recommendations when presenting the information | - Revisited recommendations and separated into ‘CoP aims’ and ‘practical recommendations’ - Presented the practical recommendations in a table, clearly identifying those which apply pre-CoP |
| Does the illustration capture our recommendations? | - We should represent the pre-CoP elements - We should capture ‘outcomes and aims’ on the map - We should remove some of the question marks on the map to illustrate that there is an end destination/goal achieved - We should keep the use of colour | - Arranged a follow up meeting with the illustrator to discuss changes and rationale |
